# Disruption of *Streptococcus mutans* and *Candida albicans* synergy by a commensal streptococcus

**DOI:** 10.1038/s41598-020-76744-5

**Published:** 2020-11-12

**Authors:** Joshua T. Huffines, Jessica A. Scoffield

**Affiliations:** Department of Microbiology, School of Medicine, University of Alabama At Birmingham, 845 19th St. South, Room 744A, Birmingham, AL 35205 USA

**Keywords:** Biofilms, Microbial communities, Fungi

## Abstract

Polymicrobial interactions in dental plaque play a significant role in dysbiosis and homeostasis in the oral cavity. In early childhood caries, *Streptococcus mutans* and *Candida albicans* are often co-isolated from carious lesions and associated with increased disease severity. Studies have demonstrated that metabolic and glucan-dependent synergism between *C. albicans* and *S. mutans* contribute to enhanced pathogenesis. However, it is unclear how oral commensals influence pathogen synergy. *Streptococcus parasanguinis*, a hydrogen peroxide (H_2_O_2_) producing oral commensal, has antimicrobial activity against *S. mutans*. In this study, we utilized a three species biofilm model to understand the impact of *S. parasanguinis* on *S. mutans* and *C. albicans* synergy. We report that *S. parasanguinis* disrupts *S. mutans* and *C. albicans* biofilm synergy in a contact and H_2_O_2_-independent manner. Further, metabolomics analysis revealed a *S. parasanguinis*-driven alteration in sugar metabolism that restricts biofilm development by *S. mutans.* Moreover, *S. parasanguinis* inhibits *S. mutans* glucosyltransferase (GtfB) activity, which is important for glucan matrix development and GtfB-mediated binding to *C. albicans* mannan. Taken together, our study describes a new antimicrobial role for *S. parasanguinis* and highlights how this abundant oral commensal may be utilized to attenuate pathogen synergism.

## Introduction

Early childhood caries (ECC) is a highly prevalent and costly biofilm-driven disease that disproportionately affects children of low socio-economic status^1,2^. *Streptococcus mutans* is commonly isolated from carious lesions of children with ECC, and as such, considered a primary etiological agent of dental caries. *S. mutans* is regarded as a highly cariogenic bacterium due to its ability to efficiently metabolize dietary carbohydrates into lactic acid, resist acid, and produce insoluble glucan, a sucrose-dependent exopolysaccharide (EPS) that is synthesized by glucosyltransferases (GTFs) and is a major virulence factor that permits the formation of robust biofilms and tooth adherence^3–7^. Although *S. mutans* is a primary cause of dental caries, oral plaque contains a consortium of microbes that interact synergistically or antagonistically^8–12^.

*Candida albicans*, an opportunistic fungal pathobiont residing throughout the body, is often co-isolated with *S. mutans* from carious lesions in children with severe ECC infections^13–15^. Several studies have reported mechanisms in which *C. albicans* and *S. mutans* display synergistic behavior. For example, *S. mutans* was found to significantly increase *C. albicans* colonization in a *Drosophila melanogaster* model. Moreover, co-infection with both species resulted in increased biofilm biomass and tooth decay compared to the single species infection in a rat caries model of infection^11,16^. Despite its involvement in ECC, *C. albicans* does not efficiently metabolize sucrose, but can readily utilize fructose and glucose^17,18^. Further, GTFs have been shown to bind to *C. albicans* mannans, thus promoting EPS production and incorporation of the fungus into the biofilm^19^. In addition, exogenous GTFs promote *C. albicans* growth^17^. Synergism between *S. mutans* and *C. albicans* has been well-documented^11,16,17,19–21^, however, the role of oral commensal streptococci in ECC microbial synergy is poorly understood.

Oral commensal streptococci are primary colonizers of the oral cavity and play a role in maintaining homeostasis by antagonizing oral pathogens via the production of hydrogen peroxide (H_2_O_2_). *Streptococcus parasanguinis*, a mitis group streptococcus and one of the most abundant (5–40% abundance) commensals on the tongue dorsum^22–25^, exhibits enhanced biofilm formation in a H_2_O_2_-dependent manner during co-culture with *Aggregatibacter actinomycetemcomitans* while simultaneously inhibiting growth of this periopathogen^26^. Furthermore, *S. parasanguinis* reduces *S. mutans* biofilm formation and pathogenesis in a rat caries model of infection in a nitrite and H_2_O_2_-dependent manner^27–29^. Due to the remarkable ability of *S. parasanguinis* to inhibit both cariogenic and periodontal pathogens, we questioned whether *S. parasanguinis* could potentially disrupt *S. mutans* and *C. albicans* synergy. Although commensal streptococci have been shown to co-aggregate and form biofilms with *C. albicans*^30^, there are no studies that dissect how streptococci, like *S. parasanguinis*, modulate the interaction between *S. mutans* and *C. albicans.* In this study, we tested the role of *S. parasanguinis* in a three species biofilm model containing *S. mutans* and *C. albicans*. Here, we report that *S. parasanguinis* significantly reduces *S. mutans* and *C. albicans* biofilm synergy. Interestingly, the reduction in *S. mutans* and *C. albicans* biofilm formation was H_2_O_2_-independent, and was the result of a disruption in *S. mutans* sucrose metabolism and a shift in the global metabolic signature by *S. parasanguinis*. Lastly, we show that *S. parasanguinis* prevents *C. albicans* from adhering to glucan and inhibits the formation of glucan by blocking GTF activity. Taken together, our study reveals a new mechanism by which *S. parasanguinis* safeguards against ECC pathogens, specifically by blocking *S. mutans* sucrose utilization, and indicates that this commensal could potentially be used as a preventative measure for oral microbial diseases.

## Results

### *Streptococcus parasanguinis *inhibits *S. mutans* and *C. albicans* biofilm synergy

**Figure 1:**
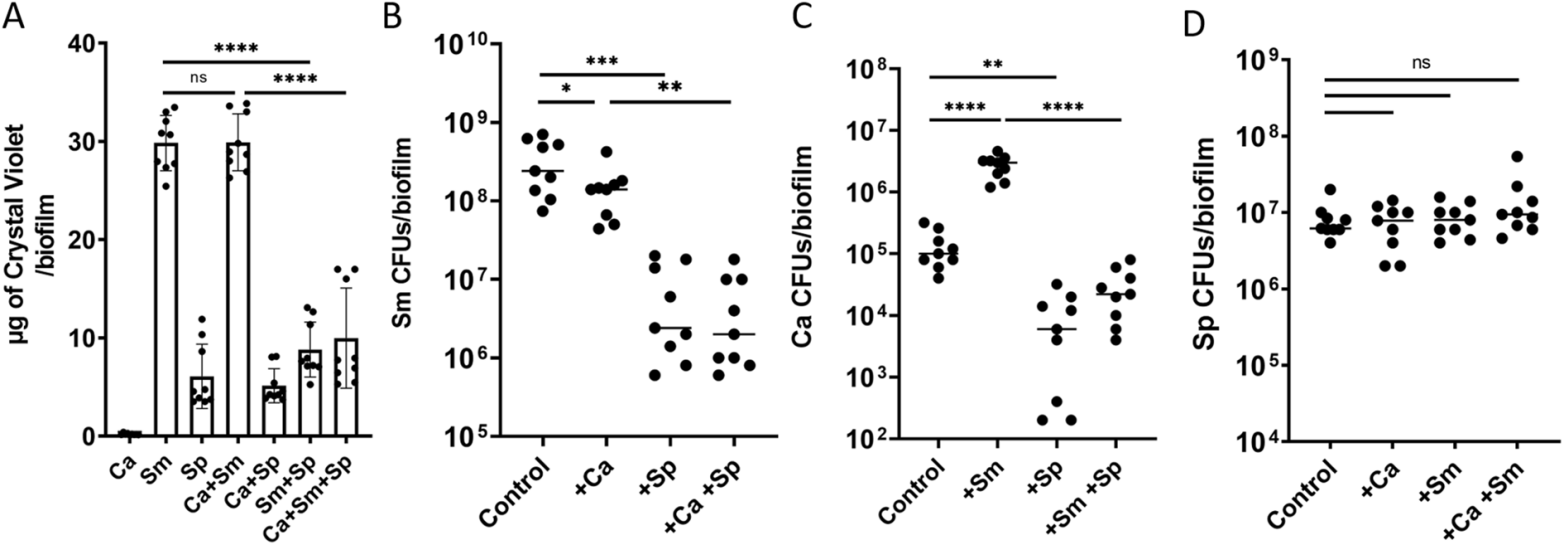
* Streptococcus parasanguinis* reduces *S. mutans* and *C. albicans* biofilm formation. (**A**) Crystal violet assay of single-, dual-, and tri-species biofilms (n = 9) ****P < 0.0001. Biofilm colony forming units (CFUs) of (**B**) *S. mutans* (Sm) *P = 0.0448 **P = 0.0015 ***P = 0.0007 (**C**) *C. albicans* (Ca) **P = 0.0012 ****P < 0.0001 (**D**) *S. parasanguinis* (Sp). All biofilms were grown in tryptic soy broth containing 0.5% yeast extract (TSBYE) and 1% sucrose for 16 h at 37 °C with 5% CO_2_. ns = not significant.

**Figure 2:**
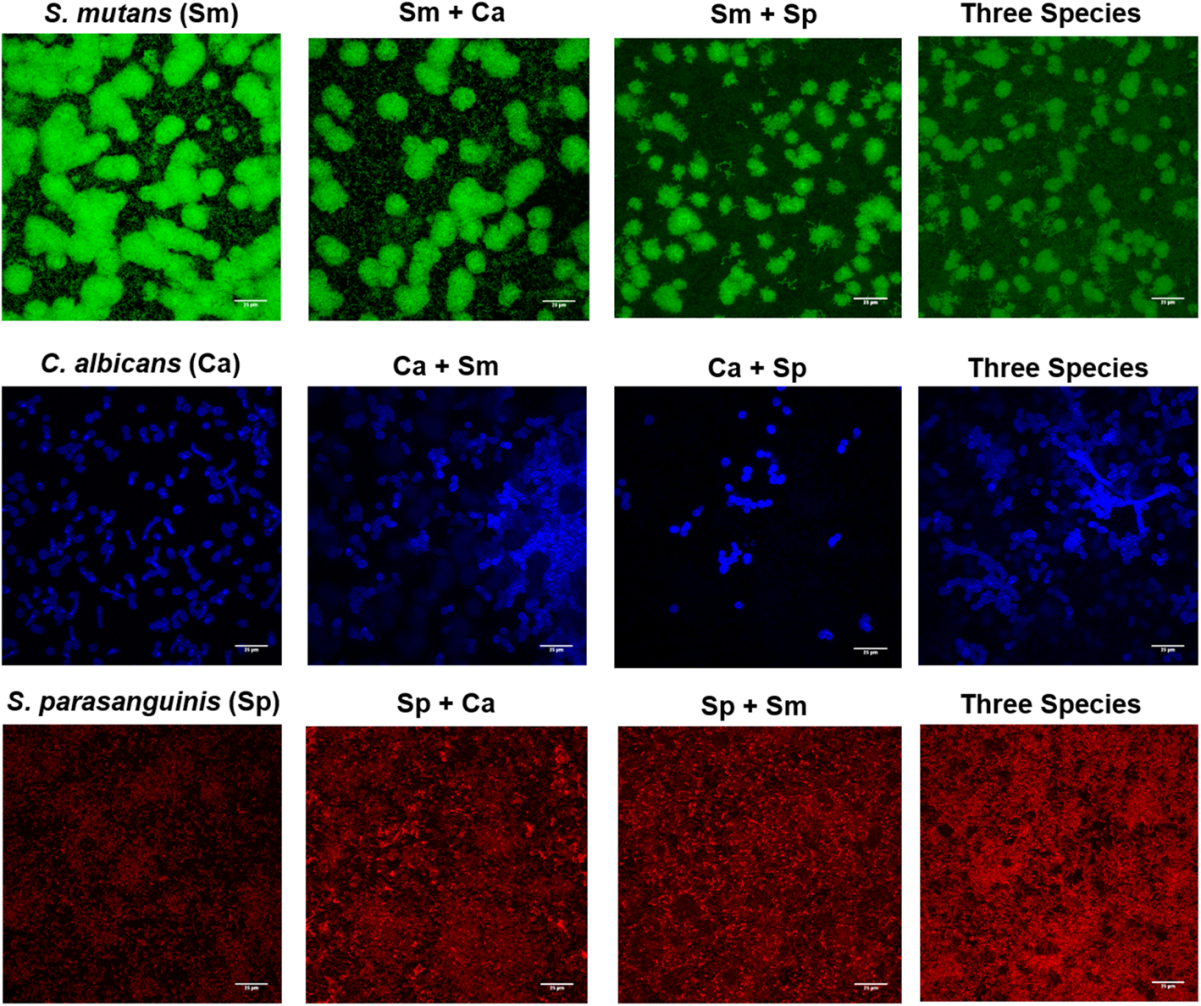
* Streptococcus parasanguinis* alters the structure of mixed biofilms. Confocal scanning laser microscopy images of wild-type *S. mutans*, *C. albicans, and S. parasanguinis* single and mixed biofilms at 60X magnification. *S. mutans* was labeled with green fluorescent protein (GFP), *C. albicans* was stained with calcofluor white, and *S. parasanguinis* was labeled with mCherry. All biofilms were grown in tryptic soy broth containing 0.5% yeast extract (TSBYE) and 1% sucrose for 16 h at 37 °C with 5% CO_2_. Scale bar: 25 µm. Images were acquired using the Nis Elements 5.0 Imaging Software (https://www.microscope.healthcare.nikon.com/products/software/nis-elements).

*Streptococcus parasanguinis* has been shown to inhibit the virulence of *S. mutans*, however, the role of this commensal streptococcus on *S. mutans* and *C. albicans* synergy is not clearly defined. In an effort to elucidate the interaction between *S. parasanguinis* and the ECC pathogens, *S. mutans* and *C. albicans*, we developed a three species biofilm model. In agreement with previous studies, crystal violet biomass quantification showed that *S. parasanguinis* and *C. albicans* produced relatively modest single species biofilms compared to the single species *S. mutans* biofilm (Fig. 1A). The dual *S. parasanguinis* and *C. albicans* biofilm was comparable to a single species *S. parasanguinis* biofilm. As expected, both the single *S. mutans* and dual *S. mutans*–*C. albicans* biofilms produced robust biofilms (Fig. 1A). Although previous studies report a synergistic increase in biofilm mass^31^, the dual-species biofilm between *C. albicans* and *S. mutans* was not significantly increased compared to the single-species *S. mutans* biofilm (*P* > 0.05; Fig. 1A). Strikingly, the addition of *S. parasanguinis* resulted in a remarkable decrease in both two (*S. mutans*) and three species biofilms (*P* < 0.0001; Fig. 1A). This result indicated the possibility that *S. parasanguinis* dominates the overall biofilm despite the presence of *S. mutans* and/or *C. albicans*. In order to quantify the abundance of each species within the biofilm we enumerated colony forming units (CFUs). Surprisingly, *S. mutans* CFUs were modestly decreased during co-culture with *C. albicans* (*P* = 0.0448; Fig. 1B). Further, there was a 2-log reduction (*P* = 0.0007) in *S. mutans* CFUs in the presence of *S. parasanguinis,* regardless of whether *C. albicans* was present (Fig. 1B). In agreement with our previous study^16^, *C. albicans* CFUs were promoted (~ 1.5 log) in the dual biofilm with *S. mutans* (*P* < 0.0001; Fig. 1C), but this was abolished when *S. parasanguinis* was added to the biofilm (*P* < 0.0001; Fig. 1C). Intriguingly, *S. parasanguinis* biomass did not change when co-cultured in single, dual, or three species biofilms (Fig. 1D). To gain insight into the overall structure of the multi-species biofilms, we used fluorescently labeled bacterial strains to visualize the biofilm architecture. Using GFP-labeled *S. mutans*, mCherry-labeled *S. parasanguinis*, and calcofluor white stained *C. albicans*, biofilm images were obtained using confocal laser scanning microscopy (CLSM). As expected, the presence of *S. parasanguinis* decreased the size of the *S. mutans* microcolonies in both the dual and three-species biofilms compared to the control biofilm (Fig. 2). Taken together, these data demonstrate *S. parasanguinis* inhibits not only *S. mutans* biofilm development, but restricts the incorporation of *C. albicans* into multi-species biofilms.

### Inhibition of *S. mutans* biofilm is H_2_O_2_ and contact-independent

**Figure 3:**
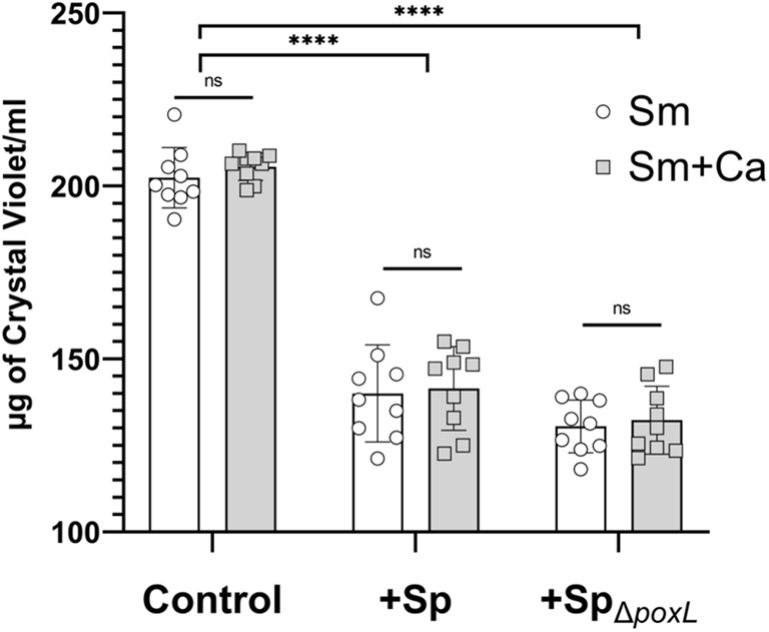
Inhibition of *S. mutans* single and dual biofilms with *C. albicans* by *S. parasanguinis* is contact and H_2_O_2_-independent. Quantification of single and dual species biofilms exposed to wild-type or pyruvate oxidase (*poxL*) deficient *S. parasanguinis* in transwells. ns = not significant; ****P < 0.0001. All biofilms were grown in tryptic soy broth containing 0.5% yeast extract (TSBYE) and 1% sucrose for 16 h at 37 °C with 5% CO_2_.

Oral commensal streptococci produce H_2_O_2_ to antagonize other bacteria^28,32–35^. The primary enzyme responsible for H_2_O_2_ production in *S. parasanguinis* is pyruvate oxidase, which is encoded by the *poxL* gene^27,28,35,36^. To investigate the potential mechanism of biofilm inhibition by *S. parasanguinis* and determine whether H_2_O_2_ production mediates this interference, both *S. mutans* and dual *C. albicans*–*S. mutans* biofilms were grown with wild-type *S. parasanguinis* or a pyruvate oxidase deficient mutant (∆ *poxL*) in transwell plates to physically separate *S. parasanguinis* from *S. mutans* and *C. albicans*. As shown in Fig. 3, the *S. mutans* single and dual (*C. albicans*) biofilms were reduced (*P* < 0.0001) at comparable levels by the presence of wild-type *S. parasanguinis* or the ∆*poxL* mutant in the transwell insert. These data indicate that the inhibition by *S. parasanguinis* was independent of physical contact and H_2_O_2_ production.

### Metabolomics reveals *S. parasanguinis*-altered sugar metabolism in multi-species biofilms

**Figure 4:**
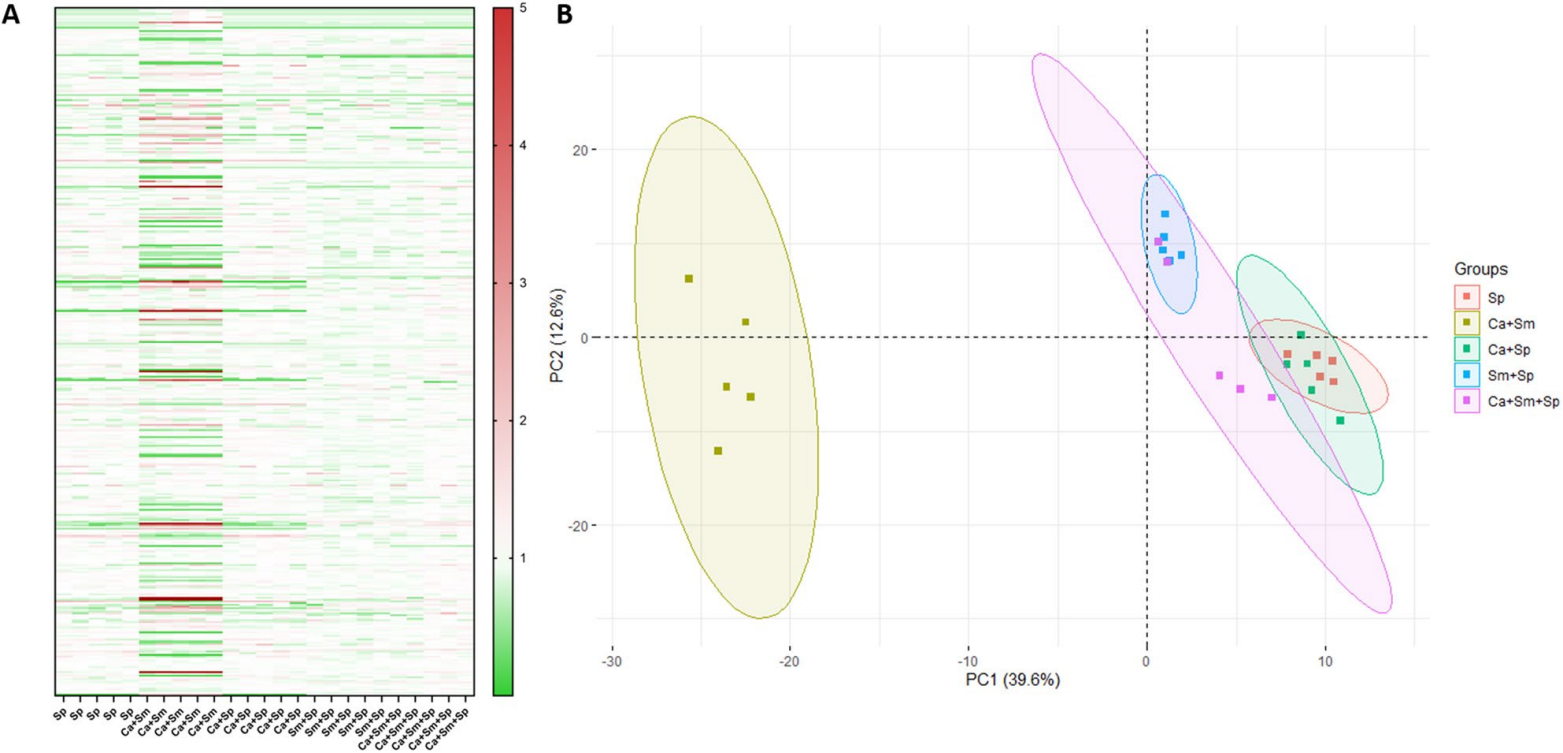
* Streptococcus parasanguinis* dominates the metabolic profile in dual- and tri-species cultures. (**A**) Global metabolomics profiling of dual- and tri-species cultures and single-species *S. parasanguinis* cultures. (**B**) Principle Component Analysis. Sp: *S. parasanguinis*; Sm: *S. mutans;* Ca: *C. albicans*. Metabolomics data were collected for 5 replicates in each group. All cultures were grown in tryptic soy broth containing 0.5% yeast extract (TSBYE) and 1% sucrose for 16 h at 37 °C with 5% CO_2_.

**Figure 5:**
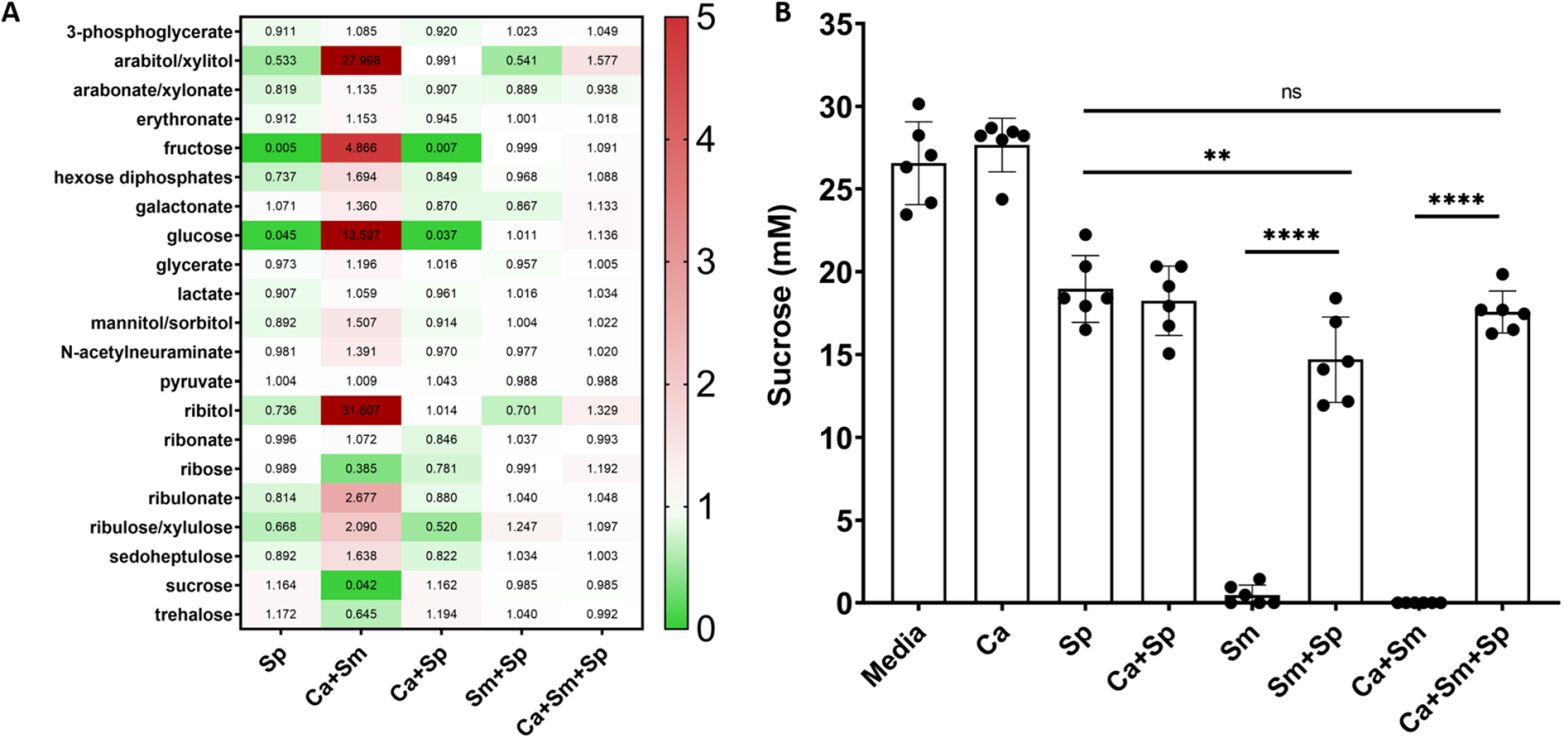
Presence of *S. parasanguinis* reduces sucrose utilization by *S. mutans* (**A**) Heatmap of carbohydrate metabolites (Value is median normalized) (**B**) Sucrose assay of single-, dual-, and tri-species cultures. Sp: *S. parasanguinis*; Sm: *S. mutans;* Ca: *C. albicans*. Metabolomics data were collected for 5 replicates in each group. ns = not significant; **P = 0.0096; ****P < 0.0001. All cultures were grown in tryptic soy broth containing 0.5% yeast extract (TSBYE) and 1% sucrose for 16 h at 37 °C with 5% CO_2_.

*Streptococcus mutans* biofilm development is driven by carbohydrate metabolism and the synthesis of a glucan matrix from sucrose^3–5,11,17,37^. To probe how *S. parasanguinis* impacts sugar metabolism, we conducted global, untargeted metabolic profiling on dual- and three-species combined planktonic and biofilm cultures as well as single species *S. parasanguinis* cultures to delineate how *S. parasanguinis* modulates the multi-species biofilm metabolome (Fig. 4A). Principle component analysis revealed the dual *C. albicans*–*S. mutans* group to be vastly different than biofilms cultured with *S. parasanguinis*, with PC1 accounting for 39.6% of the variance (Fig. 4B). Shifts in the metabolic signatures of dual and three species biofilms were largely driven by *S. parasanguinis*, as the metabolome profiles of multi-species cultures closely resembled a single species *S. parasanguinis* biofilm. The dual *C. albicans* and *S. mutans* biofilm revealed an enriched presence of broad classes of metabolites, including lipids, peptides, cofactors, and amino acids compared to single or dual species biofilms containing *S. parasanguinis*, including compounds such as tyrosol and glycerol, both of which are indicated in biofilm development for *C. albicans*^38,39^ (Figs. S1–S4). Further, the dual *C. albicans* and *S. mutans* biofilms had elevated concentrations of sugar alcohols, fructose, glucose, and fructose compared to single and dual species biofilms containing *S. parasanguinis*, (Fig. 5A), suggesting that *S. parasanguinis* potentially consumes these sugars. Notably, sucrose consumption was drastically reduced in all biofilms containing *S. parasanguinis* compared to the dual-species *S. mutans–C. albicans* biofilm. (Fig. 5A).

To confirm the altered sucrose metabolism, we quantified the sucrose concentrations from biofilm supernatants of single, dual, and three-species cultures (Fig. 5B). Compared to the un-inoculated media control, *S. parasanguinis* utilized a small fraction of sucrose, *S. mutans* single and dual biofilms with *C. albicans* utilized all of the sucrose, but *C. albicans* did not utilize sucrose when grown alone, as previously reported^17^. There was no significant difference between a single species *S. parasanguinis* or dual culture with *C. albicans* (Fig. 5B), suggesting that any sucrose consumption in these dual biofilms is solely attributed to the presence of *S. parasanguinis*. In agreement with our metabolomics findings, *S. parasanguinis* reduced sucrose utilization in the dual (*S. mutans*) and three-species biofilm cultures (*P* < 0.0001). Overall, these data show that *S. parasanguinis* drives metabolism in polymicrobial cultures and may restrict *S. mutans* and *C. albicans* synergy by blocking sucrose utilization.

### *Streptococcus parasanguinis* blocks *C. albicans* glucan binding and impairs *S. mutans* GTF activity

**Figure 6:**
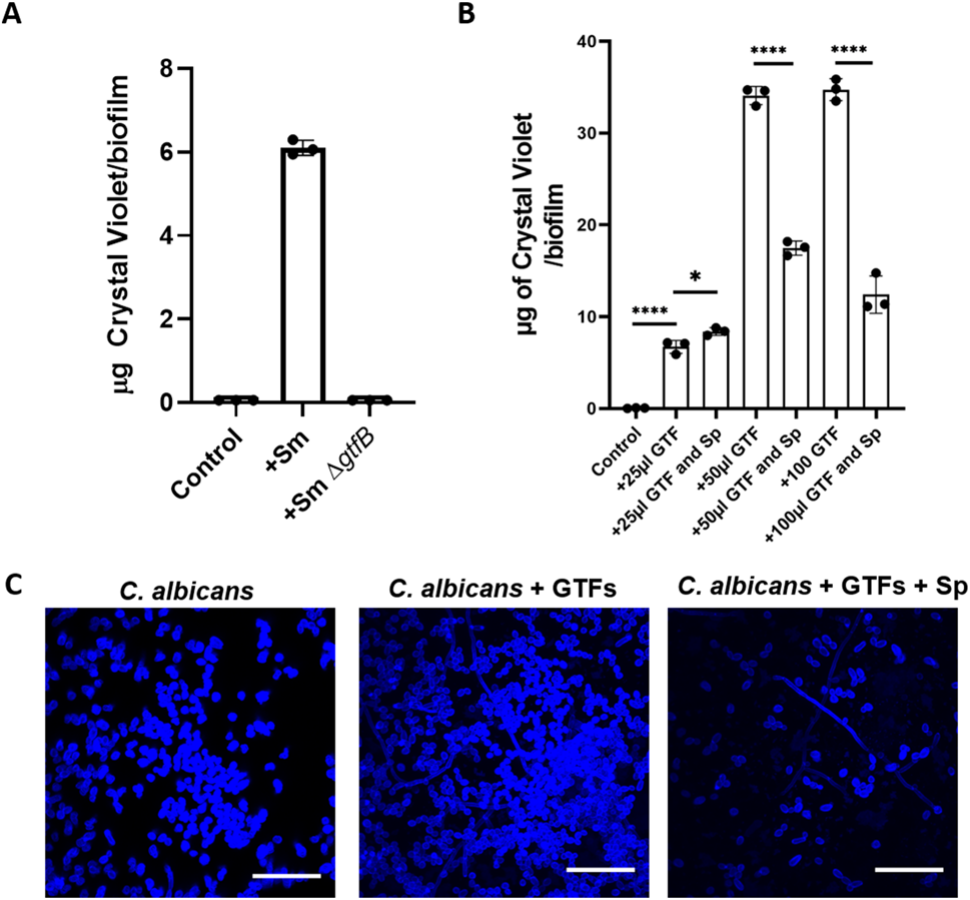
* Streptococcus parasanguinis* blocks synergy between GTFs and *C. albicans.* (**A**) Crystal violet assay of *C. albicans* biofilms with supernatant from wild-type or the *gtfB* mutant strain of *S. mutans*. (**B**) Crystal violet assay of *C. albicans* and *C. albicans*–*S. parasanguinis* biofilm with purified GTFs from overnight *S. mutans* cultures. *P = 0.0246 ****P < 0.0001; (**C**) Confocal laser scanning microscopy images of *C. albicans* biofilm stained with calcofluor white with GTFs or GTFs and *S. parasanguinis*. Scale bar: 50 µm. All cultures were grown in tryptic soy broth containing 0.5% yeast extract (TSBYE) and 1% sucrose for 16 h at 37 °C with 5% CO_2_.

GtfB-mediated glucan production and binding to *C. albicans* mannan is considered to be a central mechanism of synergy between the two species^17,19^. Therefore, we tested whether *S. parasanguinis* diminishes the ability of *C. albicans* to adhere and form biofilms with glucan that is synthesized from cell-free *S. mutans* GTFs. First, to confirm that a single *C. albicans* biofilm is indeed promoted by the addition of exogenous GTFs from *S. mutans*, we cultured *C. albicans* with ethanol precipitated supernatants from wild-type and *gtfB* deficient *S. mutans* cultures. As shown in Fig. 6A, the addition of 25 µL supernatant from wild-type *S. mutans* increased the *C. albicans* biofilm by approximately 3.5 fold compared to the *C. albicans* control (*P* < 0.0001). Supernatant harvested from the *gtfB* mutant showed defects in the ability to promote *C. albicans* biofilm compared to wild-type *S. mutans*. Next, we wanted to determine if *S. parasanguinis* blocks the ability of GTFs to enhance the *C. albicans* biofilm. To test this we added varying concentrations (25, 50, and 100 µL) of GTF supernatant to single *C. albicans* biofilms (+ /− *S. parasanguinis*). As shown in Fig. 6B, the addition of all concentrations significantly increased *C. albicans* biofilm, but *S. parasanguinis* dramatically reduced the ability of *C. albicans* to increase its biofilm for both the 50 µL and 100 µL groups (*P* < 0.0001). Finally, to verify this finding, we visualized *C. albicans* using calcofluor white and CLSM in biofilms containing 100 µL of GTFs and *S. parasanguinis*. Imaging confirmed that *S. mutans* supernatant containing exogenous GTFs do indeed promote the *C. albicans* biofilm, and this promotion is ablated by *S. parasanguinis* (Fig. 6C).

**Figure 7:**
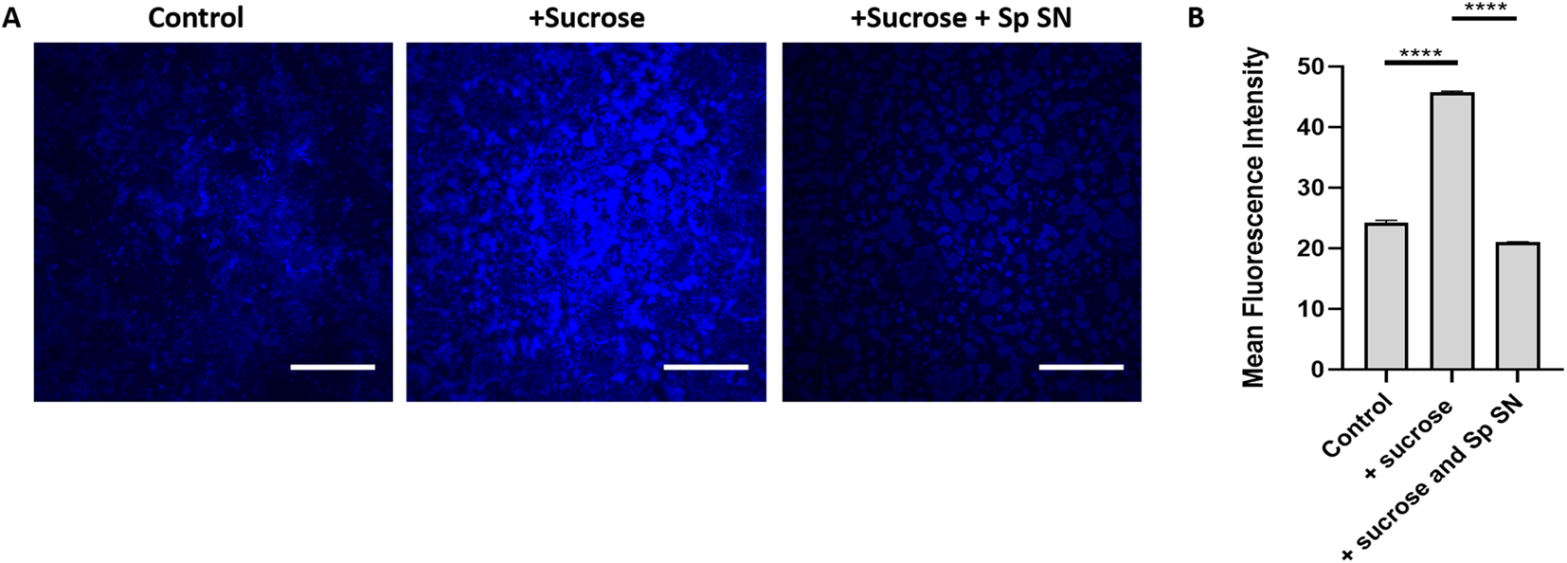
* Streptococcus parasanguinis* blocks glucan formation by GTFs. Confocal laser scanning microscopy of purified GTFs from *S. mutans* culture and cascade Blue-labeled dextran in (**A**) TSBYE, TSBYE + 1% Sucrose, or TSBYE + 1% sucrose with 100 µL of *S. parasanguinis* supernatant (SN). (**B**) Fluorescence intensity of each biofilm. ****P < 0.0001 Scale bar: 50 µm. All cultures were grown for 16 h at 37 °C with 5% CO_2_.

Due to the ability of *S. parasanguinis* to induce dramatic shifts in *S. mutans* sugar utilization, particularly sucrose, and inhibit *C. albicans* from forming optimal biofilms with GTFs, we reasoned that this result could potentially be due to impaired GTF activity by *S. parasanguinis* since GTFs convert sucrose to glucan. It is well established that glucan plays a critical role in the formation of a mature *S. mutans* biofilm and the establishment of mixed species biofilms^37,40^. To investigate whether altered sucrose utilization was driven by a reduction in GTF activity by *S. parasanguinis* we purified GTFs from the supernatant of *S. mutans* cultures. Using a Cascade blue glucan probe, we used confocal microscopy to detect modifications in the ability of purified GTFs to synthesize glucan from sucrose when *S. parasanguinis* was present. In our positive control, purified GTFs produced copious amounts of glucan with the addition of sucrose compared to GTFs that received no sucrose (negative control) (Fig. 7A). Although GTFs that did not receive sucrose produced a marginal amount of glucan compared to our positive control, we suspect this minor increase was due to carbohydrates in the growth media that permitted a small amount of glucan to be synthesized by purified GTFs. Remarkably, the addition of *S. parasanguinis*-conditioned, filtered-sterilized supernatant dramatically reduced the formation of glucan, therefore resulting in a drop in fluorescence intensity (Fig. 7A,B). *S. parasanguinis* also decreased glucan formation in single and mixed species biofilms containing *S. mutans* (Fig. S5). Overall, these data demonstrate that *S. parasanguinis* not only shifts carbohydrate metabolism during multi-species biofilm development, but can directly inhibit GTF activity through an unknown secreted factor.

## Discussion

Early childhood caries is a biofilm driven disease with *S. mutans* and *C. albicans* playing a considerable role^9,10,13,15^. Previous studies have demonstrated synergistic activity to either *S. mutans* or *C. albicans* when cultured together^11,17,19–21,40^. In addition, *C. albicans* and oral commensal streptococci have been shown to physically interact and display synergistic behavior within biofilms^30,41^, however, the impact of oral commensal streptococci in the *S. mutans*–*C. albicans* interaction is unclear. In this study, we demonstrate that *S. parasanguinis*, one of the most prominent commensals in the oral cavity^22,23^, interferes with *S. mutans* and *C. albicans* biofilm synergy in a H_2_O_2_ and contact independent manner. Supporting this observation, CLSM analysis revealed a reduction in the size of *S. mutans* microcolonies in the presence of *S. parasanguinis.* Remarkably, *S. parasanguinis* altered the global metabolic signature of the *S. mutans*–*C. albicans* cultures. Further, we provide evidence that *S. parasanguinis* blocks *C. albicans* from binding glucan, presumably by directly restricting GTF activity, which is essential for *C. albicans* binding and incorporation into the *S. mutans* biofilm, as well as robust *S. mutans* biofilm development. Numerous studies have reported that H_2_O_2_ production by commensal streptococci is the main virulence determinant that antagonizes *S. mutans*^28,35,42^. Paradoxically, we found that H_2_O_2_ had no role in the interference of *S. mutans*–*C. albicans* biofilm synergy as the level of biofilm inhibition by H_2_O_2_-deficient *S. parasanguinis* was comparable to wild-type. Overall, our studies highlight a new antimicrobial function for *S. parasanguinis* and illustrates how this commensal may potentially be used to target *S. mutans* glucan production, which is critical for not only *S. mutans* pathogenesis, but synergism with *C. albicans*.

Our global metabolomics analysis revealed that *S. parasanguinis* dominated the metabolic profile of dual and tri-species biofilms. Sucrose has consistently been recognized as the primary substrate that governs *S. mutans* virulence due to the ability of GTFs to synthesize glucan and establish a resilient biofilm^3–6,8^. *C. albicans* does not efficiently metabolize sucrose, but instead metabolizes intermediates released from the breakdown of sucrose by *S. mutans*, such as fructose and glucose^43^. In our study, fructose and glucose concentrations were elevated in the dual *S. mutans* and *C. albicans* biofilm, but the addition of *S. parasanguinis* severely reduced the levels of these substrates. Fructose, glucose, and sucrose are critical sugars in a recently proposed model of synergism between *S. mutans* and *C. albicans*^17^. Tyrosol, known for stimulating hyphae formation in *C. albicans*^38^, was found to be elevated in the *C. albicans*–*S. mutans* culture, but was largely absent from biofilms containing *S. parasanguinis* even though we observed more hyphae formation in the three species biofilms and in single species *C. albicans* cultures that were incubated with cell-free, purified GTFs. Although it is unclear as to how hyphae formation impacts synergistic biofilm formation, the GTF-dependent synergism is independent of hyphae formation^19^. Glycerol, indicated as an important regulator for biofilm formation by *C. albicans*^39^, was also higher in the dual species biofilm with *C. albicans* and *S. mutans*. One limitation of our study is that the metabolomics findings are representative of both planktonic and biofilm cultures. Although the majority of *S. mutans* cells commit to biofilm formation in the presence of sucrose rather than a planktonic mode of growth, understanding how commensals impact the metabolome of planktonic versus biofilm cells in mixed cultures would be beneficial. It is also important to note that the reduction in sugar metabolism cannot be completely explained by a reduction in *S. mutans* CFUs. *S. parasanguinis* reduces *S. mutans* CFUs by approximately 2-log. However, *S. parasanguinis* reduced *S. mutans* sucrose consumption by approximately 15-fold and inhibited the ability of GTFs to utilize sucrose in the cell-free assay when there were no *S. mutans* cells present. Overall, our findings indicate that *S. parasanguinis* may consume substrates that are essential for pathogen synergy and possibly sequester nutrients from *C. albicans*, thus making this fungus less competitive in a polymicrobial environment where it is unable to take advantage of cross-feeding from *S. mutans*.

A surprising result in our study was that *S. parasanguinis* inhibited the ability of GTFs to synthesize glucan, which could explain why *C. albicans* biofilm formation was reduced when this organism was cultured with GTF supernatant and the commensal. Alternatively, since *C. albicans* has been shown to coaggregate with oral streptococci^30,41^, *S. parasanguinis* may also obstruct the ability of GTFs to access mannans on the *C. albicans* cell surface. It is unlikely that *S. parasanguinis* and *C. albicans* compete for GTFs or glucan binding. Although *S. parasanguinis* surface adhesins have been shown to enhance biofilm formation by binding to biofilm matrix polysaccharides produced by *Pseudomonas aeruginosa*^44^, there is no evidence that demonstrate that exogenous GTFs can promote *S. parasanguinis* biofilm formation. Given that GTFs, particularly GtfB, are critical for biofilm formation, adherence of *S. mutans* to the tooth surface, and *C. albicans*–*S. mutans* synergy, the development of GtfB inhibitors is viewed as a viable strategy to target *S. mutans* pathogenesis. Structure-based screening has identified small molecules that have demonstrated efficacy against GTF activity^45–47^. Ideally, GTF targets should display selective activity against *S. mutans*, but not disturb the composition or function of the oral microbiota, especially commensals. Our findings suggest that commensal-mediated inhibition of *S. mutans* GTF activity may serve as a novel strategy to hinder *S. mutans* pathogenesis while preserving the integrity of the oral microbiome. Further dissection of the exact mechanism or molecule (s) involved in *S. parasanguinis*-mediated inhibition of *S. mutans* GTF activity is warranted and will be the focus of future studies.

To our knowledge, *S. parasanguinis* is the only oral commensal that has demonstrated broad antimicrobial and anti-biofilm effects against diverse pathogens, including cariogenic, periodontal, and respiratory pathogens. For example, the *S. parasanguinis* biofilm is promoted in a H_2_O_2_-dependent manner during co-culture with the periopathogen *A. actinomycetemcomitans*, yet this pathogen loses viability in this model^26^. In addition, *S. parasanguinis* has been shown to inhibit *S. mutans* pathogenesis in a H_2_O_2_ and nitrite-dependent manner in a rat caries model of infection^42^. Moreover, the translocation of *S. parasanguinis* into the cystic fibrosis (CF) lung has been associated with improved lung function in CF patients^48^, presumably by antagonizing the major CF pathogen *P. aeruginosa*. *S. parasanguinis* reduces viability and pathogenesis of the respiratory pathogen *P. aeruginosa *in vitro and in a *D. melanogaster* infection model via reactive nitrogenous intermediate production^34^. Lastly, a *S. parasanguinis* surface adhesin (BapA1) facilitates the enhancement of commensal biofilm formation while *P. aeruginosa* biofilm development is restricted during co-culture^44^. Taken together, these studies exhibit that the oral commensal *S. parasanguinis* has the potential to be used as a therapeutic to combat a variety of microbial infections.

In summary, this study provides new insight into how *S. parasanguinis* disrupts synergy between *S. mutans* and *C. albicans* by interfering with GTF activity. The observation that *S. parasanguinis* hinders GTF activity requires future investigation to identify the specific molecule (s) that mediates this inhibition and test whether it can be harnessed for the development of improved therapeutics. Our present study, as well as previous studies, signifies that *S. parasanguinis* is a unique commensal that may be suitable for prebiotic or probiotic use. Future studies that explore the molecular mechanisms of *S. parasanguinis* microbial antagonism will strengthen our understanding of the complexities involved in polymicrobial relationships and aid in the development commensal-derived therapies that are active against oral infections.

## Materials and methods

### Microbial strains and growth conditions

*C. albicans* SC5314, *S. mutans* UA159, *S mutans* ∆*gtfB*, *S. parasanguinis* FW213, and *S. parasanguinis* ∆*poxL*^34^ strains were used in this study. SC5314 was grown in Yeast Peptone Dextrose (YPD) whereas UA159 and FW213 were grown in Tryptic Soy Broth + 5% (w/v) Yeast Extract (TSBYE).

### Biofilm and transwell assays

Overnight cultures were grown to mid-exponential phase (OD_600_ = 0.5) and approximately 1 × 10^4^ colony forming units per milliliter (CFU/mL) were seeded into the biofilm for each species. All single, dual, and three-species biofilms were grown in TSBYE + 1% (w/v) sucrose for 16 h at 37 °C + 5% CO_2_. For all transwell experiments, a 6-well transwell insert system with a 0.4 μm pore polycarbonate membrane (Corning) was used to grow *S. parasanguinis* and the *poxL* mutant in the upper chamber and *S. mutans* and *C. albicans* biofilms in the lower well and incubated as described above. Microtiter plates were gently washed 2 times, blotted, and stained with 0.1% crystal violet. Following crystal violet staining, biofilms were washed, dried, and dissolved with 30% acetic acid as described previously^49^. To quantify biofilms to obtain values in the linear range of the Biotek plate reader, a standard curve was generated by serially diluting acetic acid dissolved biofilms and measuring the OD_562_ for each dilution. Using a standard curve, the µg of crystal violet was calculated using a trendline of the linear portion (R^2^ = 0.9966). All dilutions were measured in 96-well plates. Each assay was performed in triplicate wells and repeated three times.

### Quantification of colony forming units (CFUs)

All biofilms were gently washed with sterile PBS twice before adding 200 µL of sterile PBS for plating. The biofilms were scraped up with a 200 µL tip, vortexed for 10 s, and were serially diluted. All dilutions were plated on Todd-Hewitt Broth or blood agar plates and were incubated at 37 °C + 5% CO_2_ for a minimum of 16 h before counting.

### Confocal laser scanning microscopy (CLSM)

GFP-labeled UA159 and mCherry-labeled FW213 were used to visualize biofilms. All biofilms for confocal were grown in Ibidi µ-Slide 8 well slides (Cat #: 80826). Wells were gently washed with phosphate-buffered saline (PBS). Wells with *C. albicans* were stained with calcofluor white in PBS for 15 min before imaging. Biofilms were visualized on a Nikon A1 + confocal laser scanning microscope (CLSM) (Nikon Instruments Inc.) using a 60 × oil immersion lens. Three dimensional biofilm images were acquired using the Nis Elements 5.0 Imaging Software. All images are representative of biofilms from 3 independent experiments.

### Metabolomics analysis and sucrose quantification

Single, dual, and three species cultures were grown overnight in 20 mL of TSBYE + 1% sucrose in 50 mL conical tubes. Cells from the entire culture (planktonic and biofilm) were harvested to ensure at least 100 µL of packed cells were available for each of the 5 replicates to meet Metabolon’s processing criteria. Samples were stored in the − 80 °C freezer prior to shipment on dry ice to Metabolon, Inc (Durham, NC). All samples were processed and analyzed for raw counts by Metabolon using liquid chromatography-mass spectrometry (LCMS) methods optimized for positive ions, negative ions, and polar compounds. Raw counts provided by Metabolon were median scaled with missing values imputed with the lowest value. Principal component analysis (PCA) was calculated and graphed using R. Heatmaps of the median scaled data was made using GraphPad Prism.

To measure sucrose concentrations single, dual, and three species cultures were grown and harvested as described above for the metabolomics samples. Samples were stored overnight at − 80 °C prior to quantification. Sucrose concentrations were measured using the Glucose and Sucrose Assay Kit (Sigma Aldrich; Cat No. MAK013).

### GTF precipitation, cell-free glucan formation, and glucan quantification

*S. mutans* proteins from overnight culture supernatants were ethanol precipitated at a 1:1 ratio of 100% ethanol to precipitate extracellular *S. mutans* GTFs. The ethanol/proteins were incubated at − 80 °C for 1 h, pelleted, and re-suspended in fresh TYE (± 1% sucrose). To test the role of *S. parasanguinis* supernatant on *S. mutans* glucan formation, 100 µL of cell-free GTFs were added to 1 mL of TSBYE media that contained no sucrose, 1% sucrose, or 1% sucrose with the additional of 100 µL of filtered-sterilized spent *S. parasanguinis* media from overnight cultures. Samples were dispensed in polystyrene dishes and incubated for 16 h at 37 °C + 5% CO_2_ to permit glucan formation. For all experiments, 1 µM dextran-conjugated Cascade Blue (Molecular Probes, Invitrogen) was added to the media before overnight incubation. Fluorescence was quantified using ImageJ.

### Statistical analysis

For crystal violet, colony-forming units, and sucrose assays, we analyzed the data using Prism version 8.4.3 (GraphPad Software, LLC). An alpha value of 0.05 was used to determine statistical significance. For the metabolomics data that consisted of 5 replicates, one-way ANOVA was used to determine *P* values for the single-species group and a two-way ANOVA was used for groups with multiple species.

## Supplementary information

Supplementary Figures.
