# Supplementary material for: Disruption of Streptococcus mutans and Candida albicans synergy by a commensal streptococcus
Source: Sci Rep. 2020 Nov 12;10:19661. doi: 10.1038/s41598-020-76744-5 (PMC7661713; doi:10.1038/s41598-020-76744-5)
Supplement: Supplementary file 1 — Supplementary Figures. [file 41598_2020_76744_MOESM1_ESM.pdf]

Disruption of *Streptococcus mutans* and *Candida albicans* Synergy by a Commensal Streptococcus

Joshua T. Huffines and Jessica A. Scoffield*

Supplementary Material

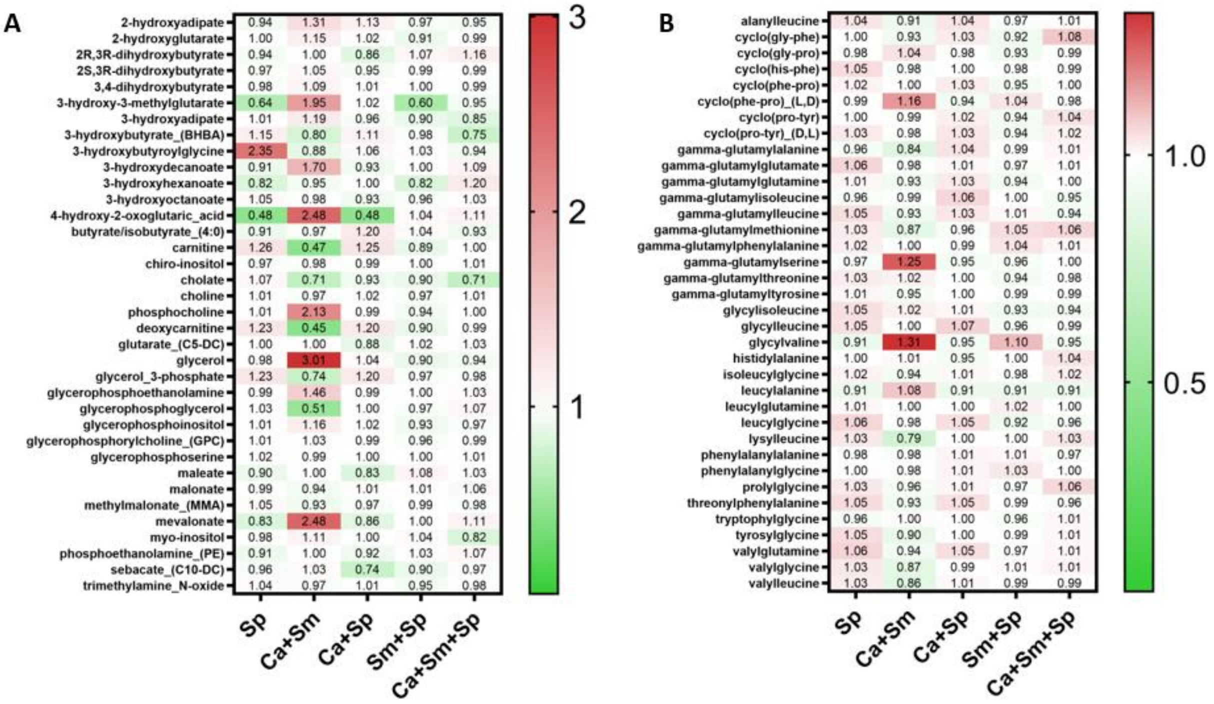

Figure S1: Heatmaps of **(A)** Lipids and **(B)** peptide metabolites. All biofilms were grown for 16 hours in TSBYE and 1% sucrose. Sp: *S. parasanguinis*; Sm: *S. mutans;* Ca: *C. albicans*. Metabolomics data were collected for 5 replicates in each group.

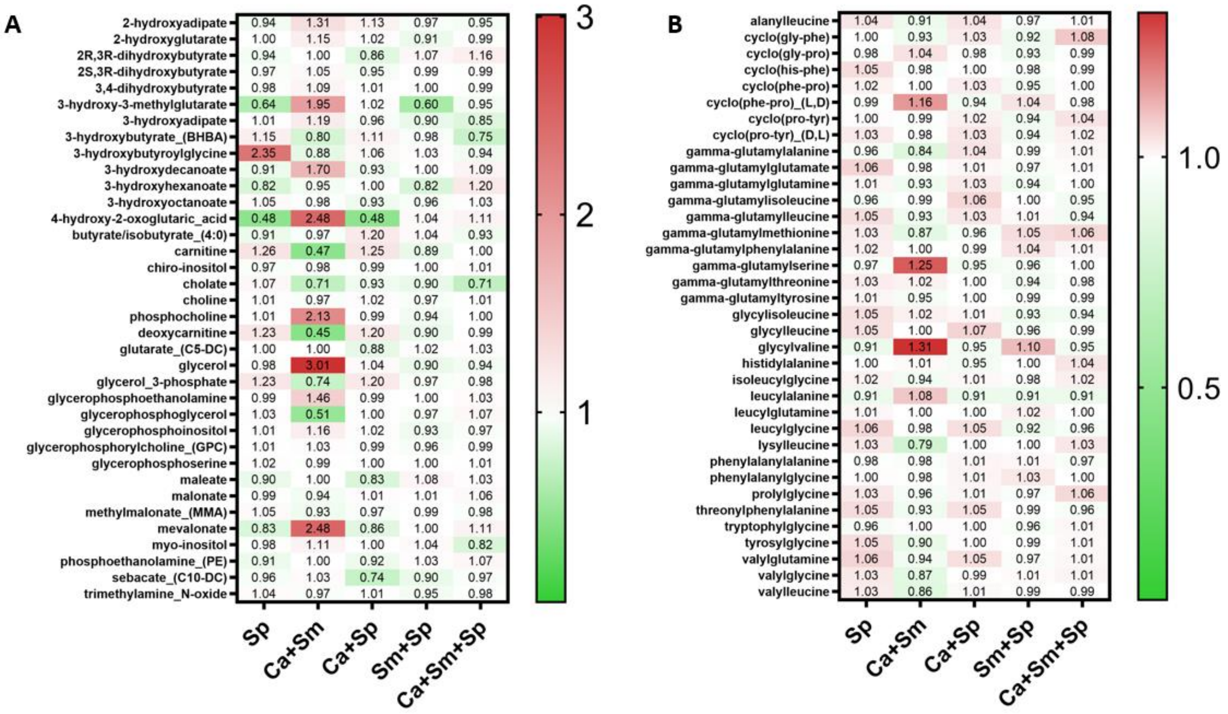

Figure S2: Heatmaps of **(A)** nucleotide and **(B)** cofactor/vitamin metabolites. All biofilms were grown for 16 hours in TSBYE and 1% sucrose. Sp: *S. parasanguinis*; Sm: *S. mutans;* Ca: *C. albicans*. Metabolomics data were collected for 5 replicates in each group.

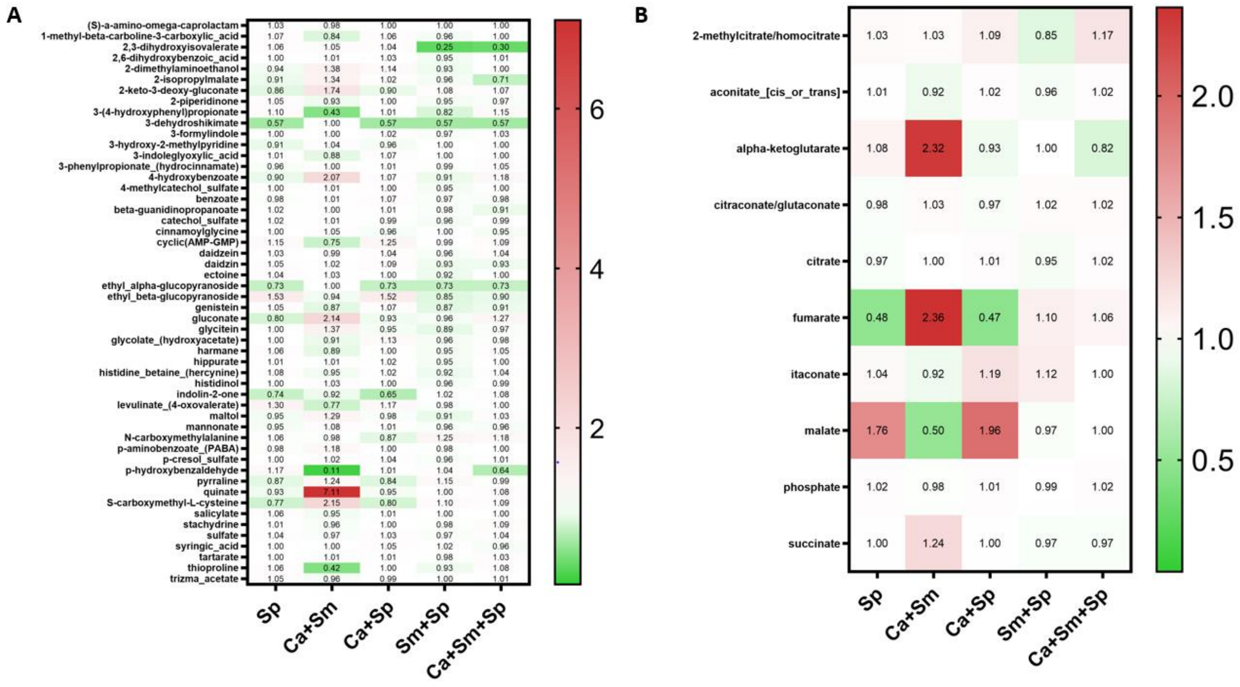

Figure S3: Heatmaps of **(A)** xenobiotic and **(B)** energy-related metabolites. All biofilms were grown for 16 hours in TSBYE and 1% sucrose. Sp: *S. parasanguinis*; Sm: *S. mutans;* Ca: *C. albicans*. Metabolomics data were collected for 5 replicates in each group.

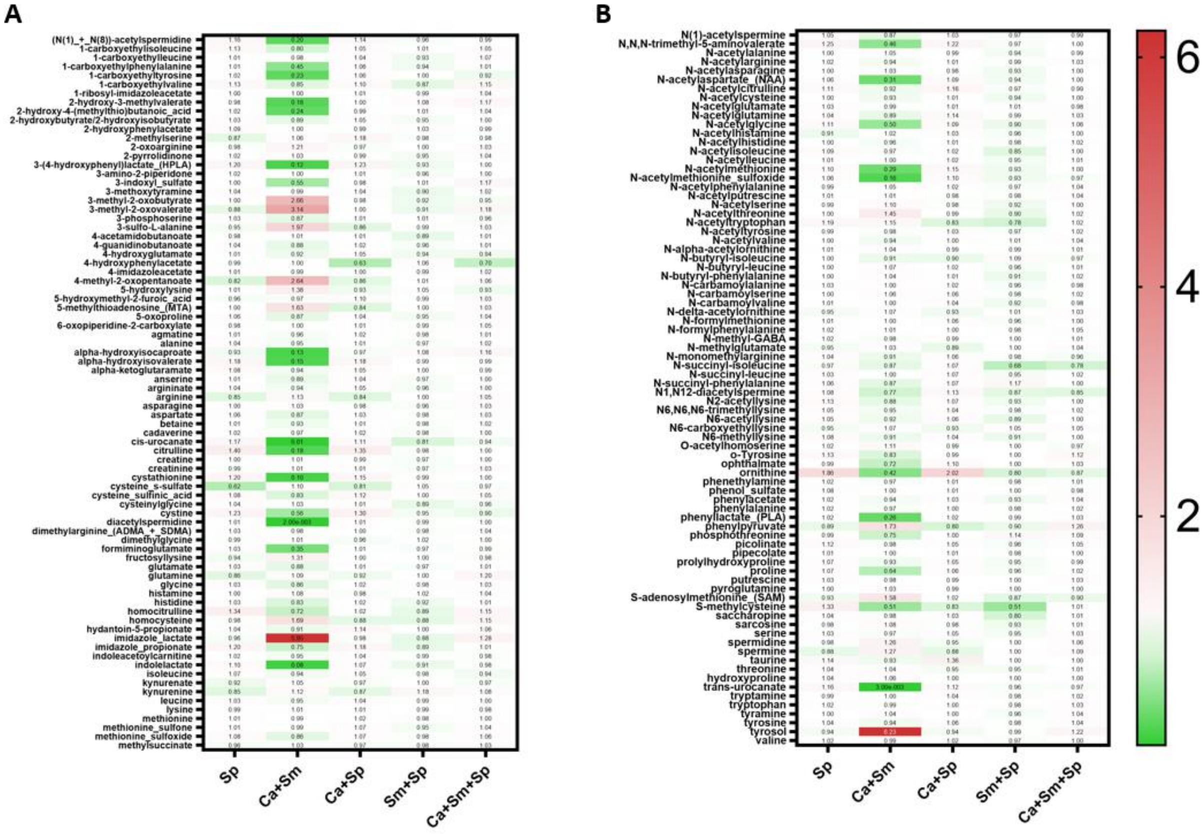

Figure S4: Heatmaps of amino acid metabolites. All biofilms were grown for 16 hours in TSBYE and 1% sucrose. Sp: *S. parasanguinis*; Sm: *S. mutans;* Ca: *C. albicans*. Metabolomics data were collected for 5 replicates in each group.

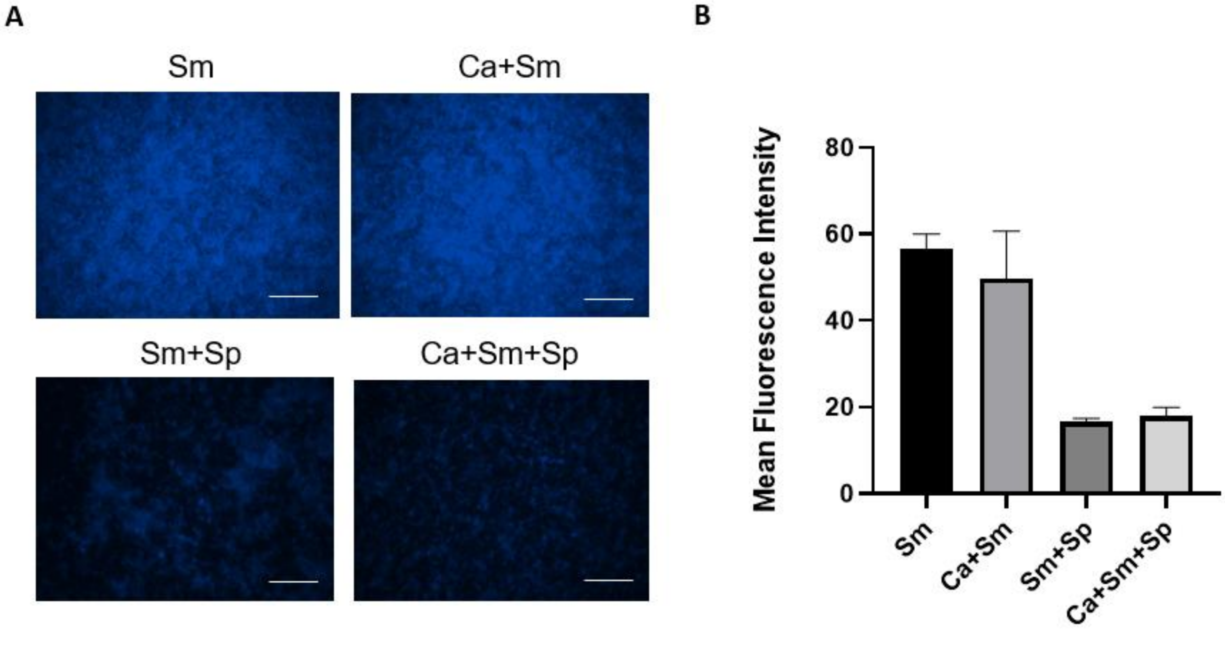

Figure S5: *S. parasanguinis* decreases glucan formation in mixed species biofilms. (A) Fluorescence microscopy images of glucan production in single-, dual-, and tri-species biofilms grown for 16 hours in TSBYE+1% sucrose. Sm: *S. mutans*, Ca: *C. albicans*, and Sp: *S. parasanguinis*. Glucan was stained with 1mM cascade Blue-labeled dextran. **(B)** Fluorescence intensity of glucan production. Scale bar: 200 µM.
